# A mathematical model of COVID-19 using fractional derivative: outbreak in India with dynamics of transmission and control

**DOI:** 10.1186/s13662-020-02834-3

**Published:** 2020-07-22

**Authors:** Amjad Salim Shaikh, Iqbal Najiroddin Shaikh, Kottakkaran Sooppy Nisar

**Affiliations:** 1 Department of Mathematics, AKI’s Poona College of Arts, Science and Commerce, Camp, Pune, India; 2 Department of Chemistry, AKI’s Poona College of Arts, Science and Commerce, Camp, Pune, India; 3 Department of Mathematics, College of Arts and Sciences, Prince Sattam bin Abdulaziz University, Wadi Aldawaser, 11991 Saudi Arabia

**Keywords:** 35A20, 35A22, 34A08, 35R11, Coronavirus, Caputo–Fabrizio derivative, Basic reproduction number, Existence and stability, Numerical simulations

## Abstract

Since the first case of 2019 novel coronavirus disease (COVID-19) detected on 30 January, 2020, in India, the number of cases rapidly increased to 3819 cases including 106 deaths as of 5 April, 2020. Taking this into account, in the present work, we have analysed a Bats–Hosts–Reservoir–People transmission fractional-order COVID-19 model for simulating the potential transmission with the thought of individual response and control measures by the government. The real data available about number of infected cases from 14 March, 2000 to 26 March, 2020 is analysed and, accordingly, various parameters of the model are estimated or fitted. The Picard successive approximation technique and Banach’s fixed point theory have been used for verification of the existence and stability criteria of the model. Further, we conduct stability analysis for both disease-free and endemic equilibrium states. On the basis of sensitivity analysis and dynamics of the threshold parameter, we estimate the effectiveness of preventive measures, predicting future outbreaks and potential control strategies of the disease using the proposed model. Numerical computations are carried out utilising the iterative Laplace transform method and comparative study of different fractional differential operators is done. The impacts of various biological parameters on transmission dynamics of COVID-19 is investigated. Finally, we illustrate the obtained results graphically.

## Introduction

Coronavirus disease is likely to emerge as a watershed moment in the history of the planet. COVID-19, the abbreviation of coronavirus disease (2019), is caused by a severe acute respiratory syndrome coronavirus 2 (SARS-CoV-2) [1], which hit the globe with a bang. In December 2019, the first outbreak was noticed in Hubei province, Wuhan, China [2]. On 30 January, 2020, the World Health Organization (WHO) revealed the COVID-19 to be a public health emergency and identified it as a pandemic on 11 March, 2020. The symptoms of COVID-19 are not specific, and many cases showed that an infected person might be asymptomatic. The majority of the cases have two common symptoms which include dry cough (68%) and fever (88%). Some of the cases have symptoms that include fatigue, muscle and joint pain, respiratory sputum production (phlegm), sore throat, loss of the sense of smell, headache or chills, and the shortness of breath. Moreover, the growth of this infection can further proceed to acute respiratory distress syndrome, severe pneumonia, and death. The COVID-19 virus spreads to large extent between people in close contact with each other (within approximately 2 m). The common incubation period ranges from 1 to 14 days [3]. In the absence of a definitive treatment modality like a vaccine, physical distancing has been accepted globally as the most efficient strategy for reducing the severity of disease and gaining control over it [4]. The concealment of physical contact in working environments, schools and other open circles is the objective of such preventive measures.

### Timeline and data analyses

On 30 January, 2020, the first case of COVID-19 was reported in India. The nation revealed its initial three cases in the state of Kerala, all were students who had a travel history from Wuhan, China [5]. The transmission escalated within March when many reported cases throughout the country were found to be connected to the people having travel history to the countries which were affected by COVID-19. On 11 March, 2020, the Indian government started taking strict actions by suspending all visas to India from 13 March, 2020 till 15 April, 2020. A 76-year-old man was the first victim of disease in the country who had returned from Saudi Arabia on 12 March, 2020 [6]. On 15 March, 2020, there were 100 confirmed cases, but this number crossed 1,000 on 28 March, 2020 and then 2,000 on 2 April, 2020 [7].

**Figure 1:**
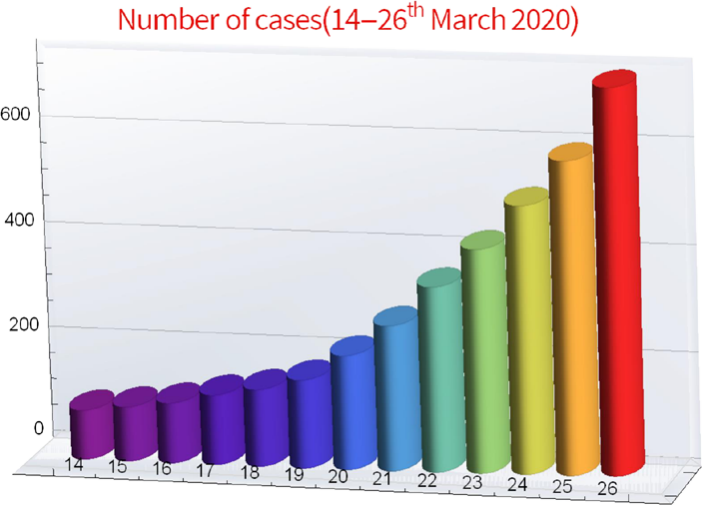
Confirmed cases of COVID-19 in India

**Figure 2:**
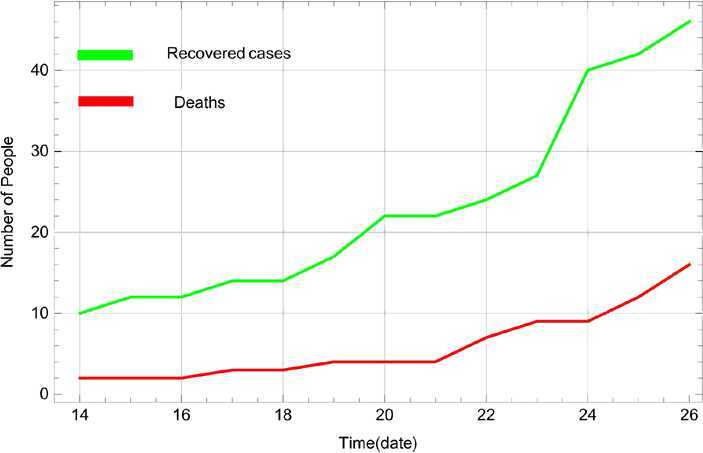
Numbers of recovered people and deaths from 14 March, 2020 to 26 March 2020 in India

The Indian government has implemented high measures for a moderate outbreak. A day-long countrywide public curfew was observed in India on 22 March, 2020. Moreover, on 24 March, 2020, the Prime Minister of India announced a countrywide lockdown for 21 days. In this study, we consider the reported cases of SARS-CoV-2, from 14 March, 2020 till 26 March, 2020, which were greater compared to initial period. We summarize the actual reported data [8] in the country as shown in the following Figs. 1 and 2.

### Mathematical model

The application of computational mathematical methods is used to simulate infections within the populations numerically. Mathematical models of infectious disease dynamics have a deep history of more than 100 years. The most common mathematical formulations are used to represent the individual transition in a community between ‘compartments’, which model the situation of individual infection to surprisingly significant accuracy. These models of compartmental disease segregate a population into groups depending on each individual’s infectious state, and related population sizes with respect to time.

In [9], Lin et al. suggested a conceptual model for the coronavirus disease 2019, which effectively catches the timeline of the COVID-19 outbreak. A mathematical model for reproducing the stage-based transmissibility of a novel coronavirus is examined by Chen et al. in [10]. In [11] Khan et al. formulated a mathematical model of coronavirus versus people, which is given by

1.1 $$ \left \{ \textstyle\begin{array}{ll} D_{t}S(t)=\Delta- \lambda S -\frac{\alpha S(I+\beta A)}{N}-\gamma SQ , \\ D_{t}E(t)=\frac{\alpha S(I+\beta A)}{N}+\gamma SQ-(1-\phi)\delta E -\phi\mu E-\lambda E, \\ D_{t}I(t)=(1-\phi)\delta E-(\sigma+\lambda)I, \\ D_{t}A(t)=\phi\mu E -(\rho+ \lambda)A, \\ D_{t}R(t)= \sigma I +\rho A -\lambda R, \\ D_{t}Q(t)= \kappa I +\nu A -\eta Q, \end{array}\displaystyle \right . $$

where *N* represents the total number of people. Further, *N* is segregated into five subclasses such as susceptible people $S(t)$, exposed people $E(t)$, infected (symptomatic), people $I(t)$, asymptotically infected $A(t)$, and the removed or the recovered people $R(t)$. The people in the reservoir or market are denoted by $Q(t)$. The description of various parameters used in this model with their values and references is given in Table 1.

**Table 1:** Fitted and referred parametric values used in the coronavirus model (1.2)

Description of parameter and notation		Value	Reference
Birth rate	Δ	53,320.19	Fitted
Contact rate	*α*	0.05	[12]
Natural mortality rate	*λ*	$\frac{1}{69.50 \times365}$	[13]
Transmission rate	*β*	0.02844	Fitted
Incubation period of *I*	*δ*	0.0717876	Fitted
Incubation period of *A*	*μ*	0.05	Fitted
The proportion of asymptomatic infection	*ϕ*	0.8243	Fitted
Disease transmission coefficient	*γ*	0.121 × 10^−7^	Fitted
Recovery or removal or rate of *I*	*σ*	0.09871	[11]
Recovery or removal or rate of *A*	*ρ*	0.854302	[11]
Contribution of the virus to *Q* by *I*	*κ*	0.000398	[11]
Contribution of the virus to *Q* by *A*	*ν*	0.001	Fitted
Removing rate of virus from *Q*	*η*	0.01	Fitted

Mathematical models, utilizing ordinary differential equations with integer-order have been used significantly for understanding the dynamics of biological systems [14, 15]. In any case, every such model depends on classical derivatives that have some limitations related to the order of differential equations under consideration. To overcome these restrictions, many authors have looked for the help of a recently emerging area of mathematics known as fractional calculus. In fractional calculus, the differential operators used are non-integer or fractional order, which possess memory properties and are valuable to demonstrate many natural phenomena, nature-related truths, and facts having nonlocal dynamics and anomalous behaviour. The study of epidemiological dynamical processes involving memory effects is appropriate because such frameworks rely on the strength of memory which is constrained by order of a fractional derivative operator.

In recent times, many researchers have developed and suggested efficient techniques to figure out real and approximate solutions of the differential equation involving fractional operators [16–22]. A lot of scholars are investigating epidemic models related to different infectious diseases involving fractional operators because they show a reasonable biphasic decline of contamination of diseases [23–27]. In the past decades, several types of fractional operators were suggested to gain better insights into the dynamics of models. Some of the commonly used operators are Riemann–Liouville, Caputo, Caputo–Fabrizio, Katugampola, Atangana–Baleanu, Hadamard and many more, where every operator has some advantages and disadvantages over the others. For example, Caputo fractional operator uses initial conditions with integer-order derivatives having clear physical meaning but has a singularity at some points. To overcome this limitation recently, Caputo and Fabrizio [28] have recommended a unique fractional derivative operator having a nonsingular and exponential kernel and what’s more, Losada and Nieto [29] examined the properties of a recently proposed fractional derivative. The advantage of this operator is that, it has a nonlocal and nonsingular kernel and best suited to describe as well as analyse the dynamics of COVID-19. For more about the Caputo–Fabrizio derivative operator, see [30–36].

We believe that a suitable mathematical model will be helpful for health officials to take positive measures to contain the spread of the contagious disease of the novel coronavirus. Motivated by this and above useful applications of the Caputo–Fabrizio (CF) operator in epidemic mathematical models, we investigate the dynamics of a novel coronavirus model based on the human-to-human transmission as well as from reservoir-to-human suggested by Khan et al. [11] in the form of a system of nonlinear differential equations. We develop the proposed model according to the characteristic of the disease and formulate it in terms of Caputo–Fabrizio fractional differential system of equations to find the condition that minimizes and controls the novel coronavirus disease spreading in the community. Finally, all the theoretical results will be verified with the help of a numerical simulation. The model is given as

1.2 $$ \left \{ \textstyle\begin{array}{ll} {}^{\mathrm{CF}}D^{\tau}_{t}S(t)=\Delta- \lambda S -\frac{\alpha S(I+\beta A)}{N}-\gamma SQ , \\ {}^{\mathrm{CF}}D^{\tau}_{t}E(t)=\frac{\alpha S(I+\beta A)}{N}+\gamma SQ-(1-\phi)\delta E -\phi\mu E-\lambda E, \\ {}^{\mathrm{CF}}D^{\tau}_{t}I(t)=(1-\phi)\delta E-(\sigma+\lambda)I, \\ {}^{\mathrm{CF}}D^{\tau}_{t}A(t)=\phi\mu E -(\rho+ \lambda)A, \\ {}^{\mathrm{CF}}D^{\tau}_{t}R(t)= \sigma I +\rho A -\lambda R, \\ {}^{\mathrm{CF}}D^{\tau}_{t}Q(t)= \kappa I +\nu A -\eta Q, \end{array}\displaystyle \right . $$

with initial conditions

1.3 $$\begin{aligned} \begin{gathered} S(0)=S_{0}\geq0,\qquad E(0)=E_{0}\geq0,\qquad I(0)=I_{0}\geq0, \\ A(0)=A_{0}\geq 0,\qquad R(0)=R_{0}\geq0,\qquad Q(0)=Q_{0}\geq0,\end{gathered} \end{aligned}$$

where *τ* is the order of CF fractional derivative operator such that $\tau\in(0,1]$.

The approximate solution and graphical results are obtained by applying the iterative Laplace transform method (ILTM).

The structure of this paper is as follows: In Sect. 2, basic definitions and results for the fractional operator and the Laplace transform are presented. In Sect. 3, an iterative scheme to find the solution of the above model using Laplace transform and new iterative method (NIM) is given; moreover, stability criteria by utilizing Banach fixed point theory along with the Picard successive approximation method are also discussed. The results involving stability analysis of the equilibria (drug-free equilibrium state and endemic equilibrium state) are presented in Sect. 4. In Sect. 5, data fitting and estimation is done, along with numerical simulations for various values of the fractional-order *τ* which are displayed graphically. Section 6 is about discussion, and finally, we give our conclusions in Sect. 7.

## Preliminaries

Some fundamental definitions and results from fractional calculus are presented in this section.

### Definition 2.1

The Caputo fractional derivative operator of order *τ* ($\tau\geq0$) and $n\in\mathbb{N} \cup\{0\}$ is defined as

2.1 $$ D^{\tau}_{t}\bigl(u(t)\bigr)= \frac{1}{\varGamma(n-\tau)} \int _{0}^{t}(t-\zeta)^{n-\tau-1} \frac{d^{n}}{dt^{n}}u(\zeta)\,d\zeta, $$

where $n-1\leq\tau< n$.

Further, the fractional-order derivative which has been considered with exponential kernel by Caputo and Fabrizio in [28] and analysed by Losada and Nieto in [29] is given by following definitions.

### Definition 2.2

Let $u\in H^{1}(a,b)$, $b > a$, $0<\tau<1 $. Then the time-fractional Caputo–Fabrizio fractional differential operator is defined by

2.2 $$ {}^{\mathrm{CF}}D^{\tau}_{t}u(t)= \frac{M(\tau)}{(1-\tau)} \int_{a}^{t}\exp \biggl[-\frac{\tau(t-\zeta)}{1-\tau} \biggr] u'(\zeta)\,d\zeta ,\quad t \geq0, 0< \tau< 1, $$

where $M(\tau)$ is a normalisation function whic depends on *τ* and satisfies $M(0)=M(1)=1$.

### Definition 2.3

The CF fractional integral operator of order $0<\tau<1$ is given by

2.3 $$ {}^{\mathrm{CF}}J^{\tau}_{t}u(t)= \frac{2(1-\tau)}{(2-\tau)M(\tau)}u(t) + \frac{2 \tau}{(2-\tau)M(\tau)} \int_{0}^{t} u(\zeta)\,d\zeta, \quad t \geq0, $$

where ${}^{\mathrm{CF}}D^{\tau}_{t}u(t)=0$, if *u* is a constant function.

### Remark 2.1

It has been observed from the previous definitions that the fractional integral of a function with order $0< \tau\leq1$ is an average between respective functions and their integral of order one. It further gives

$$ \frac{2(1- \tau)}{(2- \tau)M( \tau)}u(t) + \frac{2 \tau}{(2- \tau )M( \tau)}=1. $$

The previous equation gives an obvious formula for

$$ M( \tau)= \frac{2}{(2- \tau)}, \quad0\leq\tau\leq1. $$

### Definition 2.4

The Laplace transform (LT) for the CF fractional operator of order $0<\tau\leq1 $ for $m\in\mathbb{N}$ is given as

2.4 $$\begin{aligned} L \bigl({}^{\mathrm{CF}}D^{m+\tau}_{t}u(t) \bigr) (p)& =\frac{1}{1-\tau}L \bigl(u^{(m+1)}(t) \bigr)L \biggl(\exp \biggl( -\frac{\tau}{1-\tau}t \biggr) \biggr) \\ & = \frac{p^{m+1}L(u(t))-p^{m}u(0)-p^{m-1}u'(0)-\cdots -u^{(m)}(0)}{p+\tau(1-p)}. \end{aligned}$$

In particular, we have

$$\begin{aligned}& L \bigl({}^{\mathrm{CF}}D^{\tau}_{t}u(t) \bigr) (p) = \frac{pL(u(t))}{p+\tau (1-p)}, \quad m=0, \\& L \bigl({}^{\mathrm{CF}}D^{\tau+1}_{t}u(t) \bigr) (p) = \frac {p^{2}L(u(t))-pu(0)-u'(0)}{p+\tau(1-p)}, \quad m=1. \end{aligned}$$

## Iterative scheme and stability analysis

Consider the coronavirus model (1.2) along with initial conditions (1.3). The terms *SI*, *SA* and *SQ* in this model are nonlinear. Applying the Laplace transform on both sides of system (1.2), we obtain

3.1 $$\begin{gathered} \frac{p L(S(t))-S(0)}{p+\tau(1-p)} =L \biggl(\Delta- \lambda S -\frac{\alpha S(I+\beta A)}{N}-\gamma SQ \biggr), \\ \frac{pL(E(t))-E(0)}{p+\tau(1-p)} = L \biggl( \frac{\alpha S(I+\beta A)}{N}+\gamma SQ-(1-\phi) \delta E -\phi\mu E-\lambda E \biggr), \\ \frac{pL(I(t))-I(0)}{p+\tau(1-p)} =L\bigl((1-\phi)\delta E-(\sigma +\lambda)I\bigr), \\ \frac{pL(A(t))-A(0)}{p+\tau(1-p)} = L\bigl(\phi\mu E -(\rho+ \lambda )A\bigr), \\ \frac{pL(R(t))-R(0)}{p+\tau(1-p)} = L(\sigma I +\rho A -\lambda R), \\ \frac{pL(Q(t))-Q(0)}{p+\tau(1-p)} = L(\kappa I +\nu A -\eta Q). \end{gathered} $$

Rearranging, we get

3.2 $$\begin{aligned}& L\bigl(S(t)\bigr) =\frac{S(0)}{p}+ \biggl( \frac{p+\tau(1-p)}{p} \biggr)L \biggl(\Delta- \lambda S -\frac{\alpha S(I+\beta A)}{N}-\gamma SQ \biggr), \\& L\bigl(E(t)\bigr) = \frac{E(0)}{p}+ \biggl( \frac{p+\tau(1-p)}{p} \biggr)L \biggl( \frac{\alpha S(I+\beta A)}{N}+\gamma SQ \\& \phantom{L\bigl(E(t)\bigr) =}{} -(1-\phi)\delta E -\phi\mu E-\lambda E \biggr), \\& L\bigl(I(t)\bigr) =\frac{I(0)}{p}+ \biggl( \frac{p+\tau(1-p)}{p} \biggr)L\bigl((1-\phi)\delta E-(\sigma+\lambda)I\bigr), \\& L\bigl(A(t)\bigr) =\frac{A(0)}{p}+ \biggl( \frac{p+\tau(1-p)}{p} \biggr)L\bigl(\phi \mu E -(\rho+ \lambda)A\bigr), \\& L\bigl(R(t)\bigr) = \frac{R(0)}{p}+ \biggl( \frac{p+\tau(1-p)}{p} \biggr) L(\sigma I +\rho A -\lambda R), \\& L\bigl(Q(t)\bigr) = \frac{Q(0)}{p}+ \biggl( \frac{p+\tau(1-p)}{p} \biggr) L(\kappa I +\nu A -\eta Q). \end{aligned}$$

Further, the inverse Laplace transform of equations (3.2) yields

3.3 $$\begin{aligned}& S(t) =S(0)+L^{-1} \biggl[ \biggl( \frac{p+\tau(1-p)}{p} \biggr)L \biggl(\Delta- \lambda S -\frac{\alpha S(I+\beta A)}{N}-\gamma SQ \biggr) \biggr], \\& E(t) = E(0)+L^{-1} \biggl[ \biggl( \frac{p+\tau(1-p)}{p} \biggr)L \biggl( \frac{\alpha S(I+\beta A)}{N}+\gamma SQ \\& \phantom{E(t) =}{} -(1-\phi)\delta E -\phi\mu E-\lambda E \biggr) \biggr], \\& I(t) =I(0)+L^{-1} \biggl[ \biggl( \frac{p+\tau(1-p)}{p} \biggr)L \bigl((1-\phi)\delta E-(\sigma+\lambda)I\bigr) \biggr], \\& A(t) =A(0)+L^{-1} \biggl[ \biggl( \frac{p+\tau(1-p)}{p} \biggr)L \bigl(\phi \mu E -(\rho+ \lambda)A\bigr) \biggr], \\& R(t) = R(0)+L^{-1} \biggl[ \biggl( \frac{p+\tau(1-p)}{p} \biggr) L( \sigma I +\rho A -\lambda R) \biggr], \\& Q(t) = Q(0)+L^{-1} \biggl[ \biggl( \frac{p+\tau(1-p)}{p} \biggr) L( \kappa I +\nu A -\eta Q) \biggr]. \end{aligned}$$

The series solutions achieved by the method are given by

3.4 $$ \begin{gathered} S = \sum_{n=0}^{\infty}S_{n},\qquad E = \sum_{n=0}^{\infty}E_{n},\qquad I = \sum_{n=0}^{\infty}I_{n}, \\ A = \sum_{n=0}^{\infty}A_{n},\qquad R = \sum_{n=0}^{\infty}R_{n},\qquad Q = \sum_{n=0}^{\infty}Q_{n}.\end{gathered} $$

The nonlinearities *SI*, *SA* and *SQ* can be written as

$$ SI = \sum_{n=0}^{\infty}G_{n},\qquad SA = \sum_{n=0}^{\infty}H_{n},\qquad SQ = \sum_{n=0}^{\infty}L_{n}, $$

whereas $G_{n}$, $H_{n}$ and $L_{n}$ are further decomposed as follows:

$$\begin{aligned}& G_{n}= \sum_{i=0}^{n}S_{i} \sum_{i=0}^{n}I_{i}-\sum _{i=0}^{n-1}S_{i}\sum _{i=0}^{n-1}I_{i}, \\& H_{n}= \sum_{i=0}^{n}S_{i} \sum_{i=0}^{n}A_{i}-\sum _{i=0}^{n-1}S_{i}\sum _{i=0}^{n-1}A_{i}, \\& L_{n}= \sum_{i=0}^{n}S_{i} \sum_{i=0}^{n}Q_{i}-\sum _{i=0}^{n-1}S_{i}\sum _{i=0}^{n-1}Q_{i}. \end{aligned}$$

Using initial conditions, we get the recursive formula given by:

3.5 $$\begin{aligned}& S_{n+1}(t) = S_{n}(0)+L^{-1} \biggl[ \biggl( \frac{p+\tau (1-p)}{p} \biggr)L \biggl(\Delta- \lambda S_{n} - \frac{\alpha S_{n}(I_{n}+\beta A_{n})}{N}-\gamma S_{n}Q_{n} \biggr) \biggr], \\& E_{n+1}(t) = E_{n}(0)+L^{-1} \biggl[ \biggl( \frac{p+\tau (1-p)}{p} \biggr)L \biggl( \frac{\alpha S_{n}(I_{n}+\beta A_{n})}{N}+\gamma S_{n}Q_{n} \\& \phantom{E_{n+1}(t) =}{}-(1-\phi)\delta E_{n} -\phi\mu E_{n}-\lambda E_{n} \biggr) \biggr], \\& I_{n+1}(t) =I_{n}(0)+L^{-1} \biggl[ \biggl( \frac{p+\tau (1-p)}{p} \biggr)L\bigl((1-\phi)\delta E_{n}-(\sigma+ \lambda)I_{n}\bigr) \biggr], \\& A_{n+1}(t) =A_{n}(0)+ L^{-1} \biggl[ \biggl( \frac{p+\tau (1-p)}{p} \biggr)L\bigl(\phi\mu E_{n} -(\rho+ \lambda)A_{n}\bigr) \biggr], \\& R_{n+1}(t) =R_{n}(0)+ L^{-1} \biggl[ \biggl( \frac{p+\tau (1-p)}{p} \biggr) L(\sigma I_{n} +\rho A_{n} -\lambda R_{n}) \biggr], \\& Q_{n+1}(t) =Q_{n}(0)+ L^{-1} \biggl[ \biggl( \frac{p+\tau (1-p)}{p} \biggr) L(\kappa I_{n} +\nu A_{n} -\eta Q_{n}) \biggr]. \end{aligned}$$

### Stability analysis of the proposed method

Let $(\mathbb{B}, \parallel\cdot\parallel)$ be a Banach space with self-map *T* on $\mathbb{B}$. Also $\zeta_{n+1} = q(T,\zeta_{n})$ represents an exact recurrence formula. A fixed-point set of *T* is denoted by $U(T)$. Moreover, *T* has at least one element $\zeta_{n}$, which converges to point $x \in U(T)$. Let $\{z_{n}\in\mathbb{B}\} $ and define $j_{n} = \Vert z_{n+1}- q(T,z_{n})\Vert$. If $\lim_{n\rightarrow\infty} j^{n}=0$ implies $\lim_{n\rightarrow\infty} z^{n}=x$, then a given iteration method $\zeta_{n+1} = q(T,\zeta _{n})$ is known as *T*-stable. In this manner, this sequence $\{z_{n}\} $ has an upper bound, and the iteration is known to be the Picard’s iteration. Moreover, it is *T*-stable, if all of the above conditions are satisfied for $\zeta_{n+1} = T\zeta_{n} $.

#### Theorem 3.1

*Let*$(\mathbb{B}, \parallel\cdot\parallel)$*be a Banach space with self*-*mapTon*$\mathbb{B}$*and satisfying*

$$ \Vert T_{a}-T_{b} \Vert \leq\varGamma \Vert a-T_{a} \Vert +\varepsilon \Vert a-b \Vert $$

*for all*$a,b \in\mathbb{B}$*where*$0\leq\varGamma$, $0\leq \varepsilon<1$. *SupposeTis PicardT*-*stable*. *Consider equations* (3.5) *related to* (1.2).

#### Theorem 3.2

*Consider a self*-*mapTdefined as*

$$\begin{aligned}& T\bigl(S_{n}(t)\bigr)=S_{n+1}(t) \\& \phantom{T\bigl(S_{n}(t)\bigr)}= S_{n}(0)+L^{-1} \biggl[ \biggl( \frac{p+\tau (1-p)}{p} \biggr)L \biggl(\Delta- \lambda S_{n}-\frac{\alpha S_{n}(I_{n}+\beta A_{n})}{N}-\gamma S_{n}Q_{n} \biggr) \biggr], \\& T\bigl(E_{n}(t)\bigr)= E_{n+1}(t) \\& \phantom{T\bigl(E_{n}(t)\bigr)}= E_{n}(0)+L^{-1} \biggl[ \biggl( \frac{p+\tau (1-p)}{p} \biggr)L \biggl( \frac{\alpha S_{n}(I_{n}+\beta A_{n})}{N}\\& \phantom{T\bigl(E_{n}(t)\bigr)=}{} +\gamma S_{n}Q_{n}-(1-\phi)\delta E_{n} -\phi\mu E_{n}-\lambda E_{n} \biggr) \biggr], \\& T\bigl(I_{n}(t)\bigr)=I_{n+1}(t) = I_{n}(0)+L^{-1} \biggl[ \biggl( \frac{p+\tau (1-p)}{p} \biggr)L\bigl((1-\phi)\delta E_{n}-(\sigma+\lambda)I_{n}\bigr) \biggr], \\& T\bigl(A_{n}(t)\bigr)=A_{n+1}(t) = A_{n}(0)+ L^{-1} \biggl[ \biggl( \frac {p+\tau(1-p)}{p} \biggr)L\bigl(\phi\mu E_{n} -(\rho+ \lambda)A_{n}\bigr) \biggr], \\& T\bigl(R_{n}(t)\bigr)=R_{n+1}(t) = R_{n}(0)+ L^{-1} \biggl[ \biggl( \frac{p+\tau (1-p)}{p} \biggr) L(\sigma I_{n} +\rho A_{n} -\lambda R_{n}) \biggr], \\& T\bigl(Q_{n}(t)\bigr)=Q_{n+1}(t) = Q_{n}(0)+ L^{-1} \biggl[ \biggl( \frac{p+\tau (1-p)}{p} \biggr) L(\kappa I_{n} +\nu A_{n} -\eta Q_{n}) \biggr], \end{aligned}$$

*where*$\frac{p+\tau(1-p)}{p}$*is a Lagrange’s multiplier in fractional form*. *It is T*-*stable in*$L^{1}(a,b)$*if*

3.6 $$\begin{aligned}& \begin{gathered} \biggl(1 -\lambda F(\tau)-\frac{\alpha}{N}(K_{1}+K_{2})G( \tau )-\frac{\alpha\beta}{N}(K_{1}+K_{3})H(\tau) - \gamma(K_{1}+K_{4})J(\tau) \biggr) < 1, \\ \biggl(1+ \frac{\alpha}{N}(K_{1}+K_{2})G(\tau)+ \frac{\alpha \beta}{N}(K_{1}+K_{3})H(\tau)- \bigl((1- \phi)\delta+\phi\mu +\lambda \bigr)J_{1}(\tau) \biggr) < 1, \\ \bigl(1+ (1-\phi)\delta G_{1}(\tau) -(\sigma+ \lambda)H_{1}(\tau) \bigr) < 1, \\ \bigl( 1+\phi\mu G_{2}(\tau) -(\rho+ \lambda)H_{2}( \tau) \bigr) < 1, \\ \bigl(1+ \sigma G_{3}(\tau) +\rho H_{3}(\tau) - \lambda J_{3}(\tau) \bigr) < 1, \\ \bigl( 1+ \kappa G_{4}(\tau) +\nu H_{4}(\tau) -\eta J_{4}(\tau) \bigr) < 1.\end{gathered} \end{aligned}$$

#### Proof

The proof begins by showing that *T* has a fixed point. Therefore, for all $(m,n)\in N \times N$, we evaluate the following differences:

3.7 $$\begin{aligned}& T\bigl(S_{m}(t)\bigr)-T\bigl(S_{n}(t)\bigr)= S_{m}(t)-S_{n}(t)+ L^{-1} \biggl[ \biggl( \frac{p+\tau(1-p)}{p} \biggr)L \biggl(\Delta- \lambda S_{m} \\& \phantom{T\bigl(S_{m}(t)\bigr)-T\bigl(S_{n}(t)\bigr)=}{}-\frac{\alpha S_{m}(I_{m}+\beta A_{m})}{N}-\gamma S_{m}Q_{m} \biggr) \biggr]-L^{-1} \biggl[ \biggl( \frac{p+\tau(1-p)}{p} \biggr) \\& \phantom{T\bigl(S_{m}(t)\bigr)-T\bigl(S_{n}(t)\bigr)=}{}\times L \biggl(\Delta- \lambda S_{n}-\frac{\alpha S_{n}(I_{n}+\beta A_{n})}{N}-\gamma S_{n}Q_{n} \biggr) \biggr], \\& T\bigl(E_{m}(t)\bigr)-T\bigl(E_{n}(t)\bigr)= E_{m}(t)-E_{n}(t)+ L^{-1} \biggl[ \biggl( \frac{p+\tau(1-p)}{p} \biggr)L \biggl( \frac{\alpha S_{m}(I_{m}+\beta A_{m})}{N} \\& \phantom{T\bigl(E_{m}(t)\bigr)-T\bigl(E_{n}(t)\bigr)=}{}+\gamma S_{m}Q_{m}-(1-\phi)\delta E_{m} -\phi\mu E_{m}-\lambda E_{m} \biggr) \biggr] \\& \phantom{T\bigl(E_{m}(t)\bigr)-T\bigl(E_{n}(t)\bigr)=}{}-L^{-1} \biggl[ \biggl( \frac{p+\tau(1-p)}{p} \biggr)L \biggl( \frac {\alpha S_{n}(I_{n}+\beta A_{n})}{N}+\gamma S_{n}Q_{n} \\& \phantom{T\bigl(E_{m}(t)\bigr)-T\bigl(E_{n}(t)\bigr)=}{}-(1-\phi)\delta E_{n} -\phi\mu E_{n}-\lambda E_{n} \biggr) \biggr], \\& T\bigl(I_{m}(t)\bigr)-T\bigl(I_{n}(t)\bigr)= I_{m}(t)-I_{n}(t)+L^{-1} \biggl[ \biggl( \frac {p+\tau(1-p)}{p} \biggr)L\biggl((1-\phi)\delta E_{m} \\& \phantom{T\bigl(I_{m}(t)\bigr)-T\bigl(I_{n}(t)\bigr)=}{}-(\sigma+\lambda)I_{m}\biggr) \biggr]-L^{-1} \biggl[ \biggl( \frac{p+\tau (1-p)}{p} \biggr)L\bigl((1-\phi)\delta E_{n}-(\sigma+ \lambda)I_{n}\bigr) \biggr], \\& T\bigl(A_{m}(t)\bigr)-T\bigl(A_{n}(t)\bigr)= A_{m}(t)-A_{n}(t)+ L^{-1} \biggl[ \biggl( \frac{p+\tau(1-p)}{p} \biggr)L\bigl(\phi\mu E_{m} \\& \phantom{T\bigl(A_{m}(t)\bigr)-T\bigl(A_{n}(t)\bigr)=}{} - (\rho+ \lambda)A_{m}\bigr) \biggr] - L^{-1} \biggl[ \biggl( \frac{p+\tau (1-p)}{p} \biggr)L\bigl(\phi\mu E_{n} -(\rho+ \lambda)A_{n}\bigr) \biggr], \\& T\bigl(R_{m}(t)\bigr)-T\bigl(R_{n}(t)\bigr)= R_{m}(t)-R_{n}(t)+ L^{-1} \biggl[ \biggl( \frac{p+\tau(1-p)}{p} \biggr) L(\sigma I_{m} +\rho A_{m} \\& \phantom{T\bigl(R_{m}(t)\bigr)-T\bigl(R_{n}(t)\bigr)=}{}-\lambda R_{m}) \biggr] -L^{-1} \biggl[ \biggl( \frac{p+\tau (1-p)}{p} \biggr) L(\sigma I_{n} +\rho A_{n} -\lambda R_{n}) \biggr], \\& T\bigl(Q_{m}(t)\bigr)-T\bigl(Q_{n}(t)\bigr)= Q_{m}(t)-Q_{n}(t)+L^{-1} \biggl[ \biggl( \frac {p+\tau(1-p)}{p} \biggr) L(\kappa I_{m} +\nu A_{m} \\& \phantom{T\bigl(Q_{m}(t)\bigr)-T\bigl(Q_{n}(t)\bigr)=}{}-\eta Q_{m}) \biggr]-L^{-1} \biggl[ \biggl( \frac{p+\tau(1-p)}{p} \biggr) L(\kappa I_{n} +\nu A_{n} -\eta Q_{n}) \biggr]. \end{aligned}$$

Considering first equation of (3.7) and taking the norm of both sides of it, without loss of generality, we get

3.8 $$\begin{aligned} \bigl\Vert T\bigl(S_{m}(t)\bigr)-T\bigl(S_{n}(t) \bigr) \bigr\Vert &= \biggl\Vert S_{m}(t)-S_{n}(t) +L^{-1} \biggl[ \biggl( \frac{p+\tau(1-p)}{p} \biggr)L \biggl(\Delta- \lambda S_{m} \\ & \quad-\frac{\alpha S_{m}(I_{m}+\beta A_{m})}{N}-\gamma S_{m}Q_{m} \biggr) \biggr]-L^{-1} \biggl[ \biggl( \frac{p+\tau(1-p)}{p} \biggr) \\ &\quad\times L \biggl(\Delta- \lambda S_{n}-\frac{\alpha S_{n}(I_{n}+\beta A_{n})}{N}-\gamma S_{n}Q_{n} \biggr) \biggr] \biggr\Vert , \end{aligned}$$

so that using triangular inequality and further simplifying (3.8) yields

3.9 $$\begin{aligned} \bigl\Vert T\bigl(S_{m}(t)\bigr)-T\bigl(S_{n}(t) \bigr) \bigr\Vert &\leq \bigl\Vert S_{m}(t)-S_{n}(t) \bigr\Vert + L^{-1} \biggl[ \biggl( \frac{s+\alpha (1-s)}{s} \biggr)L \biggl[ \bigl\Vert - \lambda(S_{m}-S_{n}) \bigr\Vert \\ &\quad+ \biggl\Vert -\frac{\alpha}{N} S_{n}(I_{m}-I_{n}) \biggr\Vert + \biggl\Vert -\frac {\alpha}{N} I_{m}(S_{m}-S_{n}) \biggr\Vert \\ &\quad + \biggl\Vert -\frac{\alpha\beta}{N} A_{m}(S_{m}-S_{n}) \biggr\Vert + \bigl\Vert -\gamma S_{n}(Q_{m}-Q_{n}) \bigr\Vert \\ &\quad + \bigl\Vert -\gamma Q_{m}(S_{m}-S_{n}) \bigr\Vert + \biggl\Vert -\frac{\alpha\beta }{N} S_{n}(A_{m}-A_{n}) \biggr\Vert \biggr] \biggr]. \end{aligned}$$

As both solutions have comparative influence, we assume that

$$\begin{aligned}& \bigl\Vert S_{m}(t)-S_{n}(t) \bigr\Vert = \bigl\Vert E_{m}(t)-E_{n}(t) \bigr\Vert , \\& \bigl\Vert S_{m}(t)-S_{n}(t) \bigr\Vert = \bigl\Vert I_{m}(t)-I_{n}(t) \bigr\Vert , \\& \bigl\Vert S_{m}(t)-S_{n}(t) \bigr\Vert = \bigl\Vert A_{m}(t)-A_{n}(t) \bigr\Vert , \\& \bigl\Vert S_{m}(t)-S_{n}(t) \bigr\Vert = \bigl\Vert R_{m}(t)-R_{n}(t) \bigr\Vert , \\& \bigl\Vert S_{m}(t)-S_{n}(t) \bigr\Vert = \bigl\Vert Q_{m}(t)-Q_{n}(t) \bigr\Vert . \end{aligned}$$

Replacing this in (3.9), we get the following relation:

3.10 $$\begin{aligned} \bigl\Vert T\bigl(S_{m}(t)\bigr)-T\bigl(S_{n}(t) \bigr) \bigr\Vert &\leq \bigl\Vert S_{m}(t)-S_{n}(t) \bigr\Vert + L^{-1} \biggl[ \biggl( \frac{s+\alpha (1-s)}{s} \biggr)L \biggl[ \bigl\Vert - \lambda(S_{m}-S_{n}) \bigr\Vert \\ &\quad+ \biggl\Vert -\frac{\alpha}{N} S_{n}(S_{m}-S_{n}) \biggr\Vert + \biggl\Vert -\frac {\alpha}{N} I_{m}(S_{m}-S_{n}) \biggr\Vert \\ &\quad+ \biggl\Vert -\frac{\alpha\beta}{N} S_{n}(S_{m}-S_{n}) \biggr\Vert + \biggl\Vert -\frac{\alpha\beta}{N} A_{m}(S_{m}-S_{n}) \biggr\Vert \\ & \quad+ \bigl\Vert -\gamma S_{n}(S_{m}-S_{n}) \bigr\Vert + \bigl\Vert -\gamma Q_{m}(S_{m}-S_{n}) \bigr\Vert \biggr] \biggr]. \end{aligned}$$

Also the convergent sequences $S_{n}$, $I_{m}$, $A_{m}$ and $Q_{m}$ are bounded. Next, we can obtain five different positive constants, $K_{1}$, $K_{2}$, $K_{3}$, $K_{4}$ and $K_{5}$ for all *t* such that

3.11 $$ \Vert S_{n} \Vert < K_{1},\qquad \Vert I_{m} \Vert < K_{2},\qquad \Vert A_{m} \Vert < K_{3},\qquad \Vert Q_{m} \Vert < K_{4},\quad (m, n)\in\mathbb{N} \times \mathbb{N}. $$

Further, considering equations (3.10) and (3.11), we get

3.12 $$\begin{aligned} \bigl\Vert T\bigl(S_{m}(t)\bigr)-T \bigl(S_{n}(t)\bigr) \bigr\Vert & \leq \biggl(1 -\lambda F(\tau )- \frac{\alpha}{N}(K_{1}+K_{2})G(\tau)- \frac{\alpha\beta }{N}(K_{1}+K_{3})H(\tau) \\ &\quad-\gamma(K_{1}+K_{4})J(\tau) \biggr) \bigl\Vert (S_{m}-S_{n}) \bigr\Vert , \end{aligned}$$

where *F*, *G*, *H* and *J* are functions of $L^{-1} \{L ( \frac {p+\alpha(1-p)}{p} ) \}$. In the same manner, we can get

3.13 $$\begin{aligned} \begin{gathered} \bigl\Vert T\bigl(E_{m}(t)\bigr)-T\bigl(E_{n}(t) \bigr) \bigr\Vert \leq \biggl(1+ \frac{\alpha }{N}(K_{1}+K_{2})G( \tau)+\frac{\alpha\beta}{N}(K_{1}+K_{3})H(\tau ) \\ \phantom{\bigl\Vert T\bigl(E_{m}(t)\bigr)-T\bigl(E_{n}(t) \bigr) \bigr\Vert \leq}{}- \bigl((1-\phi)\delta+\phi\mu+\lambda \bigr)J_{1}(\tau) \biggr) \bigl\Vert (E_{m}-E_{n}) \bigr\Vert , \\ \bigl\Vert T\bigl(I_{m}(t)\bigr)-T\bigl(I_{n}(t) \bigr) \bigr\Vert \leq \bigl(1+ (1-\phi)\delta G_{1}(\tau) -( \sigma+\lambda)H_{1}(\tau) \bigr) \bigl\Vert (I_{m}-I_{n}) \bigr\Vert , \\ \bigl\Vert T\bigl(A_{m}(t)\bigr)-T\bigl(A_{n}(t) \bigr) \bigr\Vert \leq \bigl( 1+\phi\mu G_{2}(\tau ) -(\rho+ \lambda)H_{2}(\tau) \bigr) \bigl\Vert (A_{m}-A_{n}) \bigr\Vert , \\ \bigl\Vert T\bigl(R_{m}(t)\bigr)-T\bigl(R_{n}(t) \bigr) \bigr\Vert \leq \bigl(1+ \sigma G_{3}(\tau) +\rho H_{3}(\tau) -\lambda J_{3}(\tau) \bigr) \bigl\Vert (R_{m}-R_{n}) \bigr\Vert , \\ \bigl\Vert T\bigl(Q_{m}(t)\bigr)-T\bigl(Q_{n}(t) \bigr) \bigr\Vert \leq \bigl( 1+ \kappa G_{4}(\tau ) +\nu H_{4}(\tau) -\eta J_{4}(\tau) \bigr) \bigl\Vert (Q_{m}-Q_{n}) \bigr\Vert ,\end{gathered} \end{aligned}$$

where

$$\begin{aligned}& \biggl(1 -\lambda F(\tau)-\frac{\alpha}{N}(K_{1}+K_{2})G( \tau )-\frac{\alpha\beta}{N}(K_{1}+K_{3})H(\tau) - \gamma(K_{1}+K_{4})J(\tau) \biggr) < 1, \\& \biggl(1+ \frac{\alpha}{N}(K_{1}+K_{2})G(\tau)+ \frac{\alpha \beta}{N}(K_{1}+K_{3})H(\tau)- \bigl((1- \phi)\delta+\phi\mu +\lambda \bigr)J_{1}(\tau) \biggr) < 1, \\& \bigl(1+ (1-\phi)\delta G_{1}(\tau) -(\sigma+ \lambda)H_{1}(\tau) \bigr) < 1, \\& \bigl( 1+\phi\mu G_{2}(\tau) -(\rho+ \lambda)H_{2}( \tau) \bigr) < 1, \\& \bigl(1+ \sigma G_{3}(\tau) +\rho H_{3}(\tau) - \lambda J_{3}(\tau) \bigr) < 1, \\& \bigl( 1+ \kappa G_{4}(\tau) +\nu H_{4}(\tau) -\eta J_{4}(\tau) \bigr) < 1. \end{aligned}$$

Therefore, the nonlinear self-mapping *T* has a fixed point. Next, we show that *T* satisfies all the conditions in Theorem 3.1. Assuming (3.12) and (3.13) hold, we use $\varepsilon=(0,0,0,0,0,0)$ and

$$ \varGamma=\left \{ \textstyle\begin{array}{ll} (1 -\lambda F(\tau)-\frac{\alpha}{N}(K_{1}+K_{2})G(\tau )-\frac{\alpha\beta}{N}(K_{1}+K_{3})H(\tau) -\gamma(K_{1}+K_{4})J(\tau) ), \\ (1+ \frac{\alpha}{N}(K_{1}+K_{2})G(\tau)+\frac{\alpha\beta }{N}(K_{1}+K_{3})H(\tau)- ((1-\phi)\delta+\phi\mu+\lambda )J_{1}(\tau) ), \\ (1+ (1-\phi)\delta G_{1}(\tau) -(\sigma+\lambda)H_{1}(\tau) ), \\ ( 1+\phi\mu G_{2}(\tau) -(\rho+ \lambda)H_{2}(\tau) ), \\ (1+ \sigma G_{3}(\tau) +\rho H_{3}(\tau) -\lambda J_{3}(\tau) ), \\ ( 1+ \kappa G_{4}(\tau) +\nu H_{4}(\tau) -\eta J_{4}(\tau) ). \end{array}\displaystyle \right . $$

Thus, each condition in Theorem 3.2 is satisfied by the self-map *T*. Hence, *T* is Picard *T*-stable. □

## Stability analysis of the equilibria

To determine conditions for control of the novel coronavirus disease, we analyse the qualitative behaviour of the proposed model (1.2). To calculate the basic reproduction number, we start from the disease-free equilibrium by equating all variables and rate of change to zero except for $S=S_{0}$. The feasible area of the model (1.2) is given as

4.1 $$ \chi= \biggl\{ \bigl(S(t), E(t), I(t), A(t), R(t) \bigr)\in\mathbb {R}^{5}_{+}\Big| N\leq\frac{\Delta}{\lambda}, Q \in R_{+} \biggr\} . $$

The explanation given in [11] about the basic reproduction number $R_{0}$ states that it is interpreted as the expected number of secondary infections which stem from a single infected individual into an otherwise susceptible population. Moreover, it states that the disease-free equilibrium (DER) of the model (1.2) is given as

4.2 $$ E_{0}= \bigl( S^{0},0,0,0,0,0 \bigr)= \biggl( \frac{\Delta}{\lambda },0,0,0,0,0 \biggr) . $$

The behaviour of this equilibrium is studied by using linear stability analysis and we observe whether the equilibrium becomes stable, and the disease outbreak becomes under control. We analyse the dynamics of the model (1.2) around disease-free equilibrium with the help of analysis using the following results.

### Theorem 4.1

([11])

*The disease*-*free equilibrium* (*DFE*) $E_{0}$*of system* (1.2) *is locally asymptotically stable if*$R_{0} < 1$.

Moreover, the basic reproduction number denotes the maximum epidemic potential of a virus and effectively depends upon various factors like the current susceptibility of the population, whether some individuals have immunity due to prior exposure to the virus, or whether some individuals are vaccinated against the disease. Therefore, $R_{0}$ changes significantly with respect to time and estimation is based on a more realistic situation within the population. Currently, the study conducted between January 2020 and February 2020 for the 2019-nCoV virus in China by Hellewell et al. [37] suggests that the coronavirus spreads more rapidly than estimated by the World Health Organization (WHO). This work reveals that $R_{0}$ ranged between 1.4 and 6.49 with an average of 3.28 and a median of 2.79. The estimated value is very high, and it is essential to decrease it for the control of the coronavirus pandemic.

To evaluate $R_{0}$ of the model (1.2), we use the computational part given in [38]. The matrices *F* and *V* are given as

$$F= \left[ \textstyle\begin{array}{c@{\quad}c@{\quad}c@{\quad}c} 0 & \alpha& \beta\alpha& \frac{\gamma\Delta}{\lambda}\\ 0 & 0 & 0 & 0\\ 0 & 0 & 0 & 0 \\ 0 & 0 & 0 & 0 \end{array}\displaystyle \right],\qquad V= \left[ \textstyle\begin{array}{c@{\quad}c@{\quad}c@{\quad}c} \phi\mu+(1-\phi)\delta+\lambda& 0 & 0 & 0\\ (\phi-1)\delta& \lambda+\sigma& 0 & 0\\ -\phi\mu& 0 & \rho+\lambda& 0 \\ 0 & -\kappa& -\nu& \eta \end{array}\displaystyle \right]. $$

Using spectral radius, the required basic reproduction number $R_{0}$ is given as

4.3 $$ R_{0}=\frac{\mu \phi (\lambda+\sigma) (\alpha \beta \eta \lambda+\gamma \Delta \nu)+\delta (1-\phi) (\lambda+\rho) (\alpha \eta \lambda+\gamma \Delta \kappa)}{\eta \lambda (\lambda+\rho) (\lambda+\sigma) (\phi (\mu-\delta)+\delta +\lambda)}. $$

## Data fitting and numerical simulations

Here, we study numerical simulations of the CF coronavirus model (1.2). For this, we consider a few parametric values from the literature and the rest are estimated or fitted. We use the total initial population of India $N=1{,}352{,}600{,}000$ [39]. We have $N=S(0)+E(0)+I(0)+A(0)+R(0)+Q(0)$, $E_{0}=1{,}724{,}266$, $I_{0}=745$, $A_{0}=413$, $R_{0}=66$, initial susceptible population is determined to be $S_{0}=1{,}350{,}900{,}000=N-(E(0)+I(0)+A(0))-R(0)$, and $Q_{0}=10{,}000$. The life expectancy in India for the year 2019 is 69.50, so we estimate a natural mortality rate $\lambda= \frac {1}{69.50}$ per year. The birth rate is estimated as $\Delta=\frac {\lambda\times N}{365}=53{,}320.19$ and we consider this is to be the limited population in the absence of infection.

We use a set of values given in [11–13] and, estimating threshold parameters, the basic reproduction number is calculated as $R_{0}=2.58913$ for the model (1.2).

By applying ILTM successively up to four terms, we get an approximate solution of the fractional coronavirus model (1.2) in series form as given below:

$$\begin{aligned}& S(t)=1.35084\times10^{9}+59{,}227.3 \tau-2899.01 \tau^{2}+1119.34 \tau ^{3}+0.0894777 \tau^{4} \\& \phantom{S(t)=}{}+0.000387 \tau^{5}+1.230716883309\times10^{-9} t^{6} \tau^{6}+4.43058077991\times 10^{-8} \tau^{6}\\& \phantom{S(t)=}{}+t^{5} \bigl(-2.215290389957\times10^{-8} \tau^{6} -0.0000193771 \tau^{5} \bigr)\\& \phantom{S(t)=}{}+t^{4} \bigl(1.3845564937236667\times10^{-7} \tau^{6}+0.000290711 \tau^{5} \\& \phantom{S(t)=}{}+0.0111359 \tau^{4} \bigr)+t^{3} \bigl(-3.8398366759269393 \times10^{-7} \tau^{6}-0.00142145 \tau^{5} \\& \phantom{S(t)=}{}-0.119042 \tau^{4}-186.503 \tau^{3} \bigr)+t^{2} \bigl(4.873638857907262\times10^{-7} \tau^{6}+0.00271391 \tau^{5} \\& \phantom{S(t)=}{}+0.357519 \tau^{4}+1678.85 \tau^{3}-1449.43 \tau^{2} \bigr)+t \bigl(-2.6583484679494354\times10^{-7} \tau^{6} \\& \phantom{S(t)=}{}-0.00193857 \tau^{5}-0.357911 \tau^{4}-3358.03 \tau^{3}+5798.02 \tau ^{2}-59{,}227.3 \tau \bigr), \\& E(t)=1.79202\times10^{6}-66{,}189.1 \tau-212.456 \tau^{2}-0.000387843 \tau ^{5} \\& \phantom{E(t)=}{}-4.432452623242\times10^{-8} \tau^{6}+t^{5} \bigl(2.2162263116212257\times10^{-8} \tau^{6}\\& \phantom{E(t)=}{}+0.0000193836 \tau ^{5} \bigr)-1.231236839789538 \times10^{-9} t^{6} \tau^{6} \\& \phantom{E(t)=}{}+t^{4} \bigl(-1.3851414447632568\times10^{-7} \tau^{6}-0.000290807 \tau ^{5}-0.0113827 \tau^{4} \bigr) \\& \phantom{E(t)=}{}-0.0924117 \tau^{4}+t^{3} \bigl(3.8414589401434\times10^{-7} \tau^{6}+0.0014219 \tau ^{5}+0.12199 \tau^{4} \\& \phantom{E(t)=}{}+224.878 \tau^{3} \bigr)-1349.6 \tau^{3}+t^{2} \bigl(4.87569788556\times10^{-7} \tau^{6}-0.0027148 \tau ^{5} \\& \phantom{E(t)=}{}-0.36781 \tau^{4}-2024.23 \tau^{3}-106.302 \tau^{2} \bigr)+t \bigl(2.6594715\times10^{-7} \tau^{6}+0.00193 \tau^{5}\\& \phantom{E(t)=}{}+0.3696 \tau ^{4}+4048.79 \tau^{3}+424.912 \tau^{2}+66{,}189.1 \tau \bigr), \\& I(t)=21{,}239.8-19{,}384.5 \tau+ 0.0000561 t^{4} \tau^{4}+0.000674 \tau^{4}+t^{3} \bigl(11.596 \tau^{3} \\& \phantom{I(t)=}{}-0.0006742 \tau^{4} \bigr)-69.574 \tau^{3}+t^{2} \bigl(0.00236 \tau^{4}-104.362 \tau^{3}-520.381 \tau^{2} \bigr) \\& \phantom{I(t)=}{}-1040.76 \tau^{2}+t \bigl(-0.00269\tau^{4}+208.724 \tau^{3}+2081.52 \tau^{2}+19{,}384.5 \tau \bigr), \\& A(t)= 62{,}514.30-102{,}290 \tau+0.001836 t^{4} \tau^{4}+0.002203 \tau ^{4}-0.002203 t^{3} \tau^{4} \\& \phantom{A(t)=}{}+8133.38 t^{3} \tau^{3}-48{,}800.3 \tau^{3}+0.007711 t^{2} \tau^{4}-73{,}200.4 t^{2} \tau^{3}+44{,}494.3 t^{2} \tau^{2} \\& \phantom{A(t)=}{}+88{,}988.6 \tau^{2}-0.008813 t \tau^{4}+146{,}401 t \tau^{3}-177{,}977 t \tau^{2}+102{,}290t \tau, \\& R(t)=-44.1625 \tau^{2} t^{3}+ \bigl(6472.41 \tau^{2}-264.975 \tau \bigr) t^{2}+12{,}414.9 \tau^{2}+ \bigl(-25{,}094.7 \tau^{2} \\& \phantom{R(t)=}{}+25{,}785.5 \tau-264.975 \bigr) t-25{,}255.6 \tau+12{,}906.7, \\& Q(t)=-12.4041 \tau+9907.29-0.16144 t^{3} \tau^{3}+0.968642 \tau^{3}+t^{2} \bigl(1.45296 \tau^{3} \\& \phantom{Q(t)=}{}+2.37455 \tau^{2} \bigr)+4.7491 \tau^{2}+t \bigl(-2.90592 \tau^{3}-9.49821 \tau^{2}+12.4041 \tau \bigr). \end{aligned}$$

## Discussion

The considered model contains many parameters, hence several limitations arise in this study. Firstly, we have not used the detailed data of the COVID-19 for the estimation and utilized the data from the literature [11]. Besides, the parameters of population versatility were not from a precise data set. There are uncertainties in all parameters of our model, and these would translate into uncertainties in forecasts and estimates. Increasing the capacity of testing people for COVID-19 can lead to getting lucid information about the number of asymptomatic cases. This will enhance the accuracy of estimation and further progress of COVID-19. The government of India has imposed 21 days nationwide lockdown from 25 March, 2020 and was asking people to stay at home, restrict population movement, which can help limit transmission of the virus.

Tables 2 to 7 depict approximate values of all classes of model (1.2) for fractional values of $\tau=0.7,0.8,0.9,1$. Moreover, they give error analysis between approximate solution for ${\tau=1}$ of (1.2) and solution obtained from classical derivative model (1.1). Clearly, it is observed that the Caputo–Fabrizio fractional operator is highly reliable and efficient to estimate approximate solutions of mathematical models of infectious diseases.

**Table 2:** Numerical results of susceptible population $S(t)$ for fractional parameter $\tau=0.7,0.8, 0.9, 1$ and comparison between classical and approximate solution

*t*	Susceptible population *S*(*t*)				Absolute error
	*α* = 0.7	*α* = 0.8	*α* = 0.9	*α* = 1	
0	1.35088 × 10^9^	1.35088 × 10^9^	1.35088 × 10^9^	1.35088 × 10^9^	0
50	1.34057 × 10^9^	1.333653 × 10^9^	1.33500 × 10^9^	1.32537 × 10^9^	0
100	1.28180 × 10^9^	1.25053 × 10^9^	1.21088 × 10^9^	1.16185 × 10^9^	7.15256 × 10^−7^
150	1.12703 × 10^9^	1.02199 × 10^9^	8.88152 × 10^8^	7.22024 × 10^8^	0
190	9.02255 × 10^8^	6.89022 × 10^8^	4.16846 × 10^8^	7.85013 × 10^8^	0

**Table 3:** Numerical results of exposed population $E(t)$ for fractional parameter $\tau=0.7,0.8,0.9,1$ and comparison between classical and approximate solution

*t*	Exposed population *E*(*t*)				Absolute error
	*α* = 0.7	*α* = 0.8	*α* = 0.9	*α* = 1	
0	1.74512 × 10^6^	1.73824 × 10^6^	1.73129 × 10^6^	1.72427 × 10^6^	2.32831 × 10^−10^
50	1.19043 × 10^7^	1.61123 × 10^7^	2.14275 × 10^7^	2.79884 × 10^7^	1.86265 × 10^−8^
100	7.59901 × 10^7^	1.10997 × 10^7^	1.55765 × 10^8^	2.11508 × 10^8^	2.08616 × 10^−7^
150	2.51400 × 10^8^	3.72009 × 10^8^	5.26368 × 10^8^	7.19307 × 10^8^	8.34465 × 10^−7^
200	5.95356 × 10^8^	8.84518 × 10^8^	1.25536 × 10^9^	1.71808 × 10^9^	2.14577 × 10^−6^

**Table 4:** Numerical results of infectious population $I(t)$ for fractional parameter $\tau=0.7,0.8,0.9,1$ and comparison between classical and approximate solution

*t*	Infected population *I*(*t*)				Absolute error
	*α* = 0.7	*α* = 0.8	*α* = 0.9	*α* = 1	
0	7136.9	5030.55	2900.06	745	0
50	510,112	627,863	779,156	971,180	2.27708 × 10^−7^
100	2.53987 × 10^6^	3.76779 × 10^6^	5.40300 × 10^6^	7.50897 × 10^6^	9.53674 × 10^−7^
150	9.07833 × 10^6^	1.38766 × 10^7^	2.02139 × 10^7^	2.83114 × 10^7^	2.34693 × 10^−6^
200	2.31094 × 10^7^	3.54094 × 10^7^	5.15567 × 10^7^	7.20841 × 10^7^	4.57466 × 10^−6^

**Table 5:** Numerical results of asymptomatic infected population $A(t)$ for fractional parameter $\tau=0.7,0.8,0.9,1$ and comparison between classical and approximate solution

*t*	Asymptotic infected population *A*(*t*)				Absolute error
	*α* = 0.7	*α* = 0.8	*α* = 0.9	*α* = 1	
0	17,777.5	12,049.6	6959.01	413	0
50	3.42203 × 10^8^	5.00188 × 10^8^	7.00586 × 10^8^	9.48444 × 10^8^	1.19209 × 10^−7^
100	2.76018 × 10^9^	4.07858 × 10^9^	5.76149 × 10^9^	7.85341 × 10^9^	0
150	9.34626 × 10^9^	1.38584 × 10^10^	1.96297 × 10^10^	2.68150 × 10^10^	0
200	2.21928 × 10^10^	3.29629 × 10^10^	4.67521 × 10^10^	6.39332 × 10^10^	0

**Table 6:** Numerical result of recovered population $R(t)$ for fractional parameter $\tau=0.7,0.8,0.9,1$ and comparison between classical and approximate solution

*t*	Recovered population *R*(*t*)				Error
	*α* = 0.7	*α* = 0.8	*α* = 0.9	*α* = 1	
0	1311.09	647	232.73	66	0
50	1.66665 × 10^6^	2.12307 × 10^6^	2.63429 × 10^6^	3.20032 × 10^6^	1.86265 × 10^−9^
100	5.03577 × 10^6^	6.50869 × 10^6^	8.16997 × 10^6^	1.00196 × 10^7^	9.31323 × 10^−9^
150	8.07996 × 10^6^	1.05078 × 10^7^	1.32537 × 10^7^	1.63177 × 10^7^	1.49012 × 10^−8^
200	8.77051 × 10^6^	1.14705 × 10^7^	1.45318 × 10^7^	1.79540 × 10^7^	3.35276 × 10^−8^

**Table 7:** Numerical results of reservoir population $Q(t)$ for fractional parameter $\tau=0.7,0.8,0.9,1$ and comparison between classical and approximate solution

*t*	Reservoir population *Q*(*t*)				Absolute error
	*α* = 0.7	*α* = 0.8	*α* = 0.9	*α* = 1	
0	9901.27	9900.9	9900.68	9900.61	0
15	10,042.4	10,166.6	10,194.8	10,216.9	0
30	9992.84	9777.12	9447.94	8986.48	5.45697 × 10^−12^
45	8357.09	7058.65	5276.77	2940.08	2.27394 × 10^−11^
60	4105.23	337.37	0	0	4.18368 × 10^−11^

**Figure 3:**
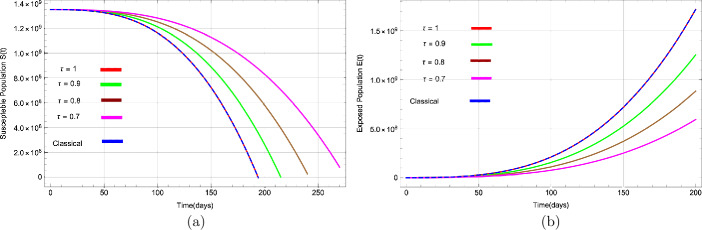
Dynamical behavior of (**a**) suspected population $S(t)$ and (**b**) exposed population $E(t)$ for various values of *τ* with respect to time (days)

**Figure 4:**
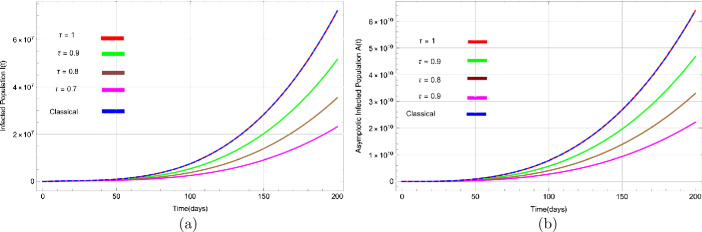
Dynamical behavior of (**a**) infected (symptomatic) population $I(t)$ and (**b**) asymptotic population $A(t)$ for various values of *τ* with respect to time (days)

**Figure 5:**
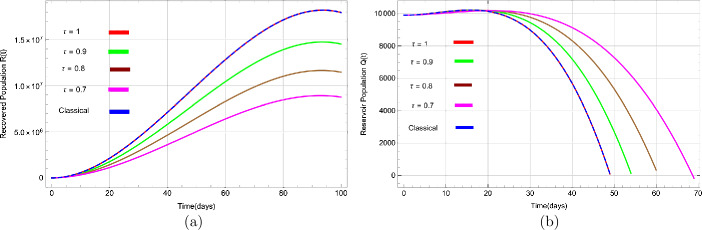
Dynamical behavior of (**a**) recovered population $R(t)$ and (**b**) reservoir population $Q(t)$ for various values of *τ* with respect to time (days)

Figures 3(a) to 5(b) show the behaviour of susceptible people $S(t)$, exposed people $E(t)$, infected people $I(t)$, asymptotic infected people $A(t)$, recovered people $R(t)$, and people in reservoir $Q(t)$ versus time *t* in days, respectively, for distinct values of *τ*.

Figure 3(a) demonstrates that the number of susceptible people decreases rapidly and converges to zero as the value of *τ* decreases. The graph in Fig. 3(b) for exposed people shows that as the value of *τ* goes down, the rate of increase also reduces. Figure 4(a) shows that the infected population increases sharply with non-integer values of *τ*, but as the value of *τ* decreases, the rate of increase of infection gets lower. It also shows that at a very slow pace ($\tau=0.1$), the number of infected people is significantly lower.

The number of asymptomatic infected people $A(t)$ also increases for various values of *τ*, as shown in Fig. 4(b). Likewise, it can be seen in Fig. 5(a) that people are recovered or removed (dead) very rapidly with a change of *τ*. Finally, Fig. 5(b) depicts people in the market or reservoir, which decreases with fractional values of *τ*. We have also plotted solutions obtained by classical derivative in Figs. 3(a) to 5(b) by a blue dashed line to compare with approximate solutions using fractional derivative and noted that both solutions are almost identical.

**Figure 6:**
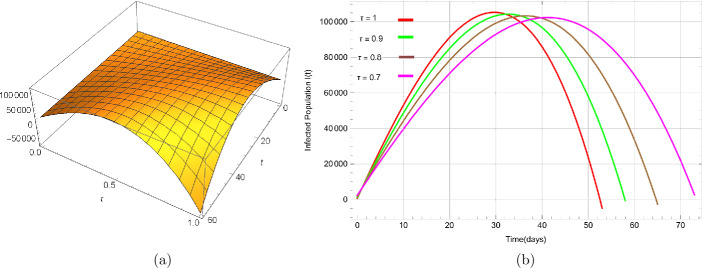
Dynamical behavior of the infected population $I(t)$ where (**a**) shows the surface for $0<\tau\leq1$ and $0\leq t \leq60$ while (**b**) shows infected population with parameters $\mu=0.08$, $\sigma=0.0237$

Figure 6(a) shows a surface plot of infected people with respect to time *t* ($0\leq t \leq60$) and *τ* ($0 < \tau\leq1$). From Fig. 6(b), it is observed that the rate of infectious population with different fractional-order parameter given by *τ* significantly depends upon parameters. The proportion of asymptomatic infection ($\phi=0.9543$), recovery or removal rate of *I* ($\sigma=0.0237$) and incubation period of the asymptomatic population ($\mu=0.08$). The size of the infectious population significantly reduces compared to Fig. 4(a) by fitting these parametric values appropriately. Moreover, the infection rate also depends on the people in the reservoir or market ($Q(t)$), and making this class near to zero reduces the infection rapidly. The simulations carried out justified our control strategies to minimize the infected population and reservoir. Moreover, they also revealed that a difference in the esteem influences the dynamics of the epidemic. The non-integer order has a notable effect on the dynamics of the epidemic, and this model depends continuously on the time-fractional derivative.

To the early end of the COVID-19 pandemic and in the absence of some sure treatment like a vaccine, preventive measures to reduce the spread of the virus are recommended. Some of such precautions include social distancing, decreasing number of contacts of susceptible population, mitigation, containment, suppression against the infection and self-quarantine of entire population living in affected areas are crucial. Moreover, the policy of reducing the transmission period by finding and isolating patients as quickly as possible through efforts by the quarantine authorities and complete participation of the public would benefit greatly the control of this infection.

## Conclusions

In this paper, we proposed the pandemic problem of the COVID-19. We formulated a fractional mathematical model to suggest some possible control strategies that will be helpful for public health officials to eradicate this contagious disease from the community. It has been observed from the present work that infectious diseases can be effectively modelled with nonlocal Caputo–Fabrizio fractional derivative operator. Moreover, by implementing the Banach fixed point theory, the stability criteria for steady solutions and existence have been verified. Approximate solutions and graphical demonstration by using iterative Laplace transform method of the CF fractional coronavirus model have been presented. It is noted that memory features in CF derivative explore hidden dynamics of the infection in the mathematical models of infectious disease, which is not possible to realise with integer-order derivatives.

It is to be noted from this analysis that one of the very key parameters is the disease transmission coefficient *γ* which plays a significant role in determining the basic reproduction number $R_{0}$. The control measures suggest that the infection will be eradicated rapidly once the control strategies will be implemented in a true manner. In the end, all the theoretical results are supported with the help of graphical and tabular representation by using mathematical software. This model becomes highly reliable when real-time and actual estimates of transmission structures are available.

In the future study, the dynamics of COVID-19 pandemic along with the effect of some control measures by including more classes into the present model will be proposed. These compartments include symptomatic but not traced population, asymptomatic and quarantined individuals. The model will be an extended version of the present model and proposed using memory features and nonlocality.
